# The Association of Blood Glucose Levels and Arterial Stiffness (Cardio-Ankle Vascular Index) in Patients With Type 2 Diabetes Mellitus

**DOI:** 10.7759/cureus.20408

**Published:** 2021-12-14

**Authors:** Yazid A Alghamdi, Faisal S Al-Shahrani, Saif S Alanazi, Fahad A Alshammari, Abdullah M Alkhudair, Noor-Ahmed Jatoi

**Affiliations:** 1 Internal Medicine, King Fahd Hospital of the University, Imam Abdulrahman Bin Faisal University, Al Khobar, SAU

**Keywords:** diabetes mellitus type 2, cavi, cardiovascular prevention, atherosclerosis, arterial stiffness

## Abstract

Introduction: Diabetes mellitus causes a major burden on healthcare systems all around the world. It has been documented that type 2 diabetes mellitus (T2DM) is associated with long-term vascular complications including micro-vascular, macro-vascular, and mixed-vascular disorders. Several studies have concluded that the increment of arterial wall stiffness is correlated with an increase in the risk of cardiovascular adverse events and the mortality associated with it.

Aims: This study purposed to evaluate the arterial stiffness measurements, using Cardio-Ankle Vascular Index (CAVI), in T2DM patients, and the relationship with the fasting blood glucose (FBG), hemoglobin A1c (HbA1c), and other factors that may increase the risk of elevated arterial stiffness in T2DM patients.

Methodology: A total of 200 patients were recruited from the outpatient setting at King Fahd Hospital of the University, Al Khobar. A total of 181 patients fit the inclusion criteria. The charts of the patients who fit the inclusion criteria were reviewed and data related to age, gender, body mass index, smoking history, FBG, HbA1c levels, blood pressure (mmHg) measurements, and CAVI scores were collected.

Results: The elevation in CAVI readings was noted to be more prominent in the senior age group. Hypertensive patients also showed a significant increase in CAVI readings. In addition, higher CAVI readings were more associated with the male gender rather than females. All of which showed a significant correlation. Furthermore, although it was not significant, higher FBG levels and HbA1c readings were correlated with higher CAVI readings.

Conclusion: The results of the study suggest that factors like age, smoking status, gender, and the increase in blood pressure as well as the increase in blood glucose levels are correlated with higher CAVI readings in T2DM patients. This demonstrates their important effect on arterial wall stiffness while showing that CAVI can be used in predicting the prognosis of arterial wall health in patients with diabetes.

## Introduction

Arterial stiffness is independently associated with the increased risk of cardiovascular events. It is affected by a wide variety of factors such as age, gender, smoking, body mass index (BMI), and multiple metabolic and cardiovascular factors. Lifestyle and habits like smoking were found to be associated with a higher incidence of arterial stiffness [1]. In addition, several chronic conditions are also associated with arterial stiffness. One of the most important metabolic disorders is type 2 diabetes mellitus (T2DM), which has deleterious consequences on the whole body system. The cardiovascular system is no exception as both macrovascular and microvascular involvements are major complications in patients suffering from T2DM. These complications account for around 70% of deaths in people with T2DM as they have a four times higher risk of developing serious cardiovascular events [2].

Arterial stiffness can be measured using different means; cardio-ankle vascular index (CAVI) is a new means of arterial wall stiffness assessment that can detect changes in arterial wall stiffness shortly after changes in circulation happen. It measures arterial wall stiffness in several different arteries. It can be calculated by recording the distance from the level of the aortic valve to a different measuring site and the time delay between the closure of the aortic valve to the detected difference in arterial pressure wave at the target measurement site. The distinctive features in CAVI are that it changes in a short time period in response to changes in the circulation, and it reflects the condition of smooth muscles contraction rather than the blood pressure (BP). So, it is not affected by the changes in BP levels [2,3].

Recent studies have demonstrated that changes in CAVI are associated with plasma glucose levels. It is higher in patients who have diabetes when compared with subjects that do not have it. Since the disturbance in the glucose levels in diabetic patients can bring about vascular complications, which may result in serious cardiovascular events, this study is conducted to review the correlation between arterial stiffness assessed by CAVI with fasting blood glucose (FBG) and hemoglobin A1c (HbA1c) levels in patients with T2DM [4].

## Materials and methods

Subjects

A total of 200 patients were approached and 188 patients achieved the inclusion criteria. Therefore, they were included in the study. All patients were diagnosed with T2DM for five years or more. The study collected data regarding FBG, HbA1c, and lipid profile as total cholesterol, low-density lipoprotein (LDL), high-density lipoprotein (HDL), and triglycerides (TRG) were noted at the time of CAVI measurement ± three months. We also considered the history of hypertension and dyslipidemia based on whether they are diagnosed with hypertension or dyslipidemia or not in the King Fahd Hospital of the University (KFHU) database.

The recent diagnostic criteria were published by the American Diabetes Association (ADA), which documented that diabetes disease is confirmed when a patient has an FBG of 126 mg/dL or more, HbA1c of 6.5% or more, two hours postprandial blood glucose of 200 mg/dL or more, or any patient with classical symptoms of hyperglycemia or hyperglycemic crisis with random plasma glucose of 200 mg/dL or more [5]. According to the Japan Society for Vascular Failure, CAVI readings below 8 are considered normal, CAVI readings equal to 8 and less than 9 are considered borderline, and CAVI readings more than 9 are considered abnormal [6].

Inclusion Criteria

Patients with T2DM who satisfied the aforementioned ADA diagnostic criteria of T2DM and attended the Diabetic Care Centre at KFHU were included in the study. In addition, they must have been diagnosed with T2DM for at least five years.

Exclusion Criteria

Patients who did not meet the ADA criteria for T2DM were excluded from the study. Patients who have been diagnosed with diabetes for less than five years and those who have been diagnosed with other types of diabetes were excluded.

Sample size and sampling technique

The data of 200 patients were collected from the medical records stored in the KFHU database (QuadraMed). A total of 188 patients met the inclusion criteria and 12 were excluded. A patient was considered diabetic if found and documented in the database as being diabetic, and all patients who met the inclusion criteria were included in this study.

The data collection sheet was created by the study authors. It included the patient’s demographic data, risk factors of T2DM including smoking, hypertension, and dyslipidemia, brachial and ankle CAVI measurements for both right and left sides, and laboratory results involving HbA1c, FBG, and lipid profile.

Data analysis

Data gathering was established in Microsoft Excel (Microsoft Corporation, Redmond, WA) and was analyzed using Statistical Package for the Social Sciences (SPSS) version 23 (IBM SPSS Statistics, Armonk, NY) with a 95% confidence interval. The quantitative variables were reported using average and standard deviation. Qualitative variables were recorded by using frequency and percentage. The study analysis utilized cross-tabulation and graphs. For inferential statistics, the normality of the data was tested first by using the Shapiro-Wilk test. Also, the Mann-Whitney U test was used to compare means in the comparison of dichotomous variables. For correlation between two continuous variables, Spearman’s correlation test was used. One-way analysis of variance (ANOVA) was also applied to compare means between three groups or more. The level of significance was set at 0.05.

## Results

A total of 188 participants were included in this study. The results show that the average age of the study participants was 54.99 years. The average body weight was 89.1 kg and the average height was 163.47 cm. Furthermore, the results reveal that the average BMI is more towards the obese side with an average score of 33.29 kg/m^2^. In regards to gender, the majority of the study participants were males (61.1%) compared to the females who make up 38.9% of the study sample. Regarding BMI, it can be noted that 9.0% of the study participants were in the normal BMI range while 30.3% of the participants were overweight, whereas the obese participants make up the majority of the sample (60.6%). In terms of smoking, most of the participants were smokers (73.8%) compared to 26.2% non-smokers. Moreover, 64.3% of the study participants were diagnosed with hypertension. And in regards to dyslipidemia, a total of 137 (72.8%) of the participants have dyslipidemia, compared to 37.2% who do not. Table 1 presents the sociodemographic characteristics of the study participants.

**Table 1:** Sociodemographic characteristics of participants (n = 188).

Characteristics	Average ± SD
Age	54.99 ± 11.4
Weight	89.1 ± 19.67
Height	163.47 ± 9.14
BMI	33.29 ± 7.33
Characteristics	Frequency (percentage)
Gender	
Male	115 (61.1%)
Female	73 (38.9%)
BMI category	
Normal	17 (9.04%)
Overweight	57 (30.31%)
Obese	114 (60.63%)
Smoking	
Yes	136 (73.8%)
No	52 (26.2%)
Hypertension	
Yes	121 (64.3%)
No	67 (35.7%)
Dyslipidemia	
Yes	137 (72.8%)
No	51 (37.2%)

**Figure 1:**
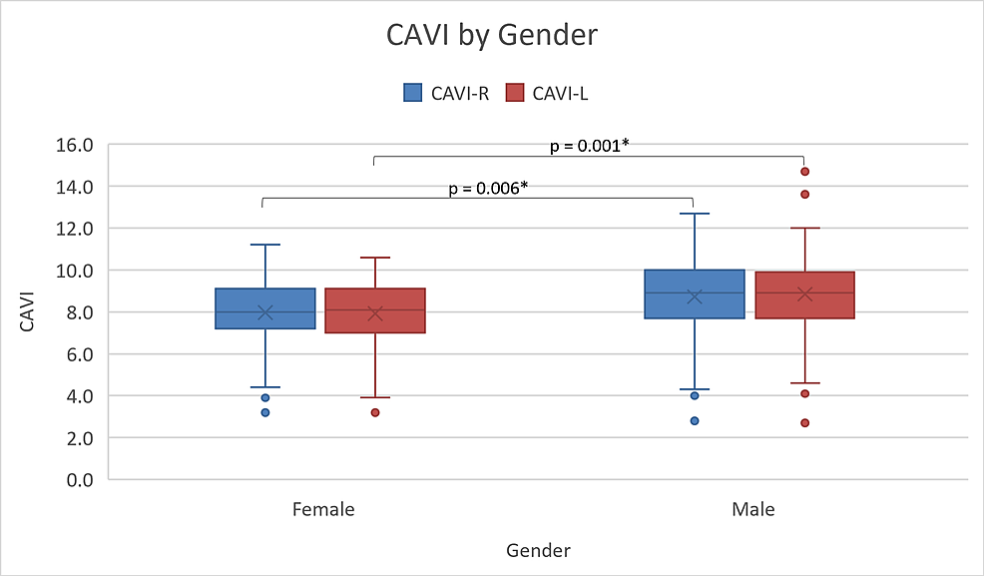
Box and whiskers plots of right and left CAVI averages by gender. CAVI: cardio-ankle vascular index; CAVI-R: right cardio-ankle vascular index; CAVI-L: left cardio-ankle vascular index. * P-value of 0.05 or less is statistically significant.

**Figure 2:**
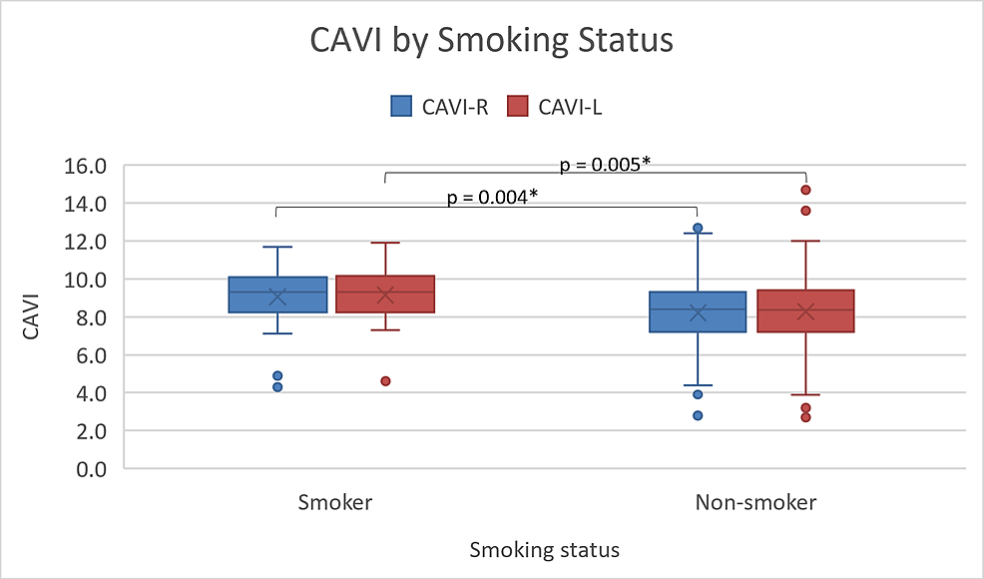
Box and whiskers plot of right and left CAVI by smoking status. CAVI: cardio-ankle vascular index; CAVI-R: right cardio-ankle vascular index; CAVI-L: left cardio-ankle vascular index. * P-value of 0.05 or less is statistically significant.

**Figure 3:**
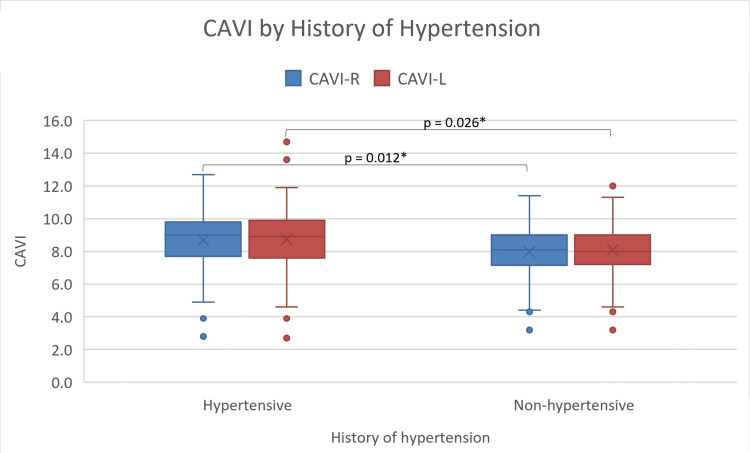
Box and whiskers plot of right and left CAVI by the history of hypertension CAVI: cardio-ankle vascular index; CAVI-R: right cardio-ankle vascular index; CAVI-L: left cardio-ankle vascular index. * P-value of 0.05 or less is statistically significant.

Regarding the scores of CAVI, the results reveal that the male CAVI scores are significantly (p = 0.001) higher than female scores with male and female CAVI mean scores of 8.81 ± 1.8 and 7.88 ± 1.6, respectively (Figure 1). The results further demonstrate that the average CAVI scores for patients who smoke (9.1 ± 1.5) are higher than those of non-smokers (8.35 ± 1.8), with a p-value of 0.03 (Figure 2). In addition, hypertensive participants (8.69 ± 1.79) show higher CAVI scores compared to non-hypertensive ones (8.00 ± 1.71; p = 0.01) (Figure 3). Also, participants who are considered normal and overweight have higher average CAVI scores (9.2 ± 1.96 and 9.08 ± 1.56, respectively) than those of the obese participants (8.09 ± 1.82) with a p-value < 0.001. Table 2 compares the average CAVI scores for the sample characteristics.

**Table 2:** Results of CAVI average scores according to the selected variables (n = 188). * P-value of 0.05 or less is statistically significant.

Variable	Average ± SD	p-value
Gender		
Male	8.81 ± 1.8	0.001^*^
Female	7.88 ± 1.6	
BMI		
Normal	9.2 ± 1.96	0.001^*^
Overweight	9.08 ± 1.56	
Obese	8.09 ± 1.82	
Smoking status		
Smoker	9.1 ± 1.5	0.03^*^
Non-smoker	8.35 ± 1.8	
Hypertension		
Yes	8.69 ± 1.79	0.01^*^
No	8.00 ± 1.71	
Dyslipidemia		
Yes	8.53 ± 1.91	0.37
No	8.26 ± 1.39	

**Figure 4:**
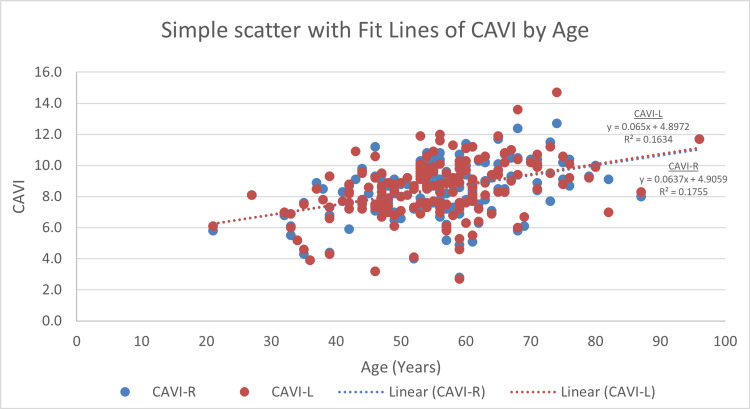
Scatter plot of right and left CAVI scores by age. CAVI: cardio-ankle vascular index; CAVI-R: right cardio-ankle vascular index; CAVI-L: left cardio-ankle vascular index.

**Figure 5:**
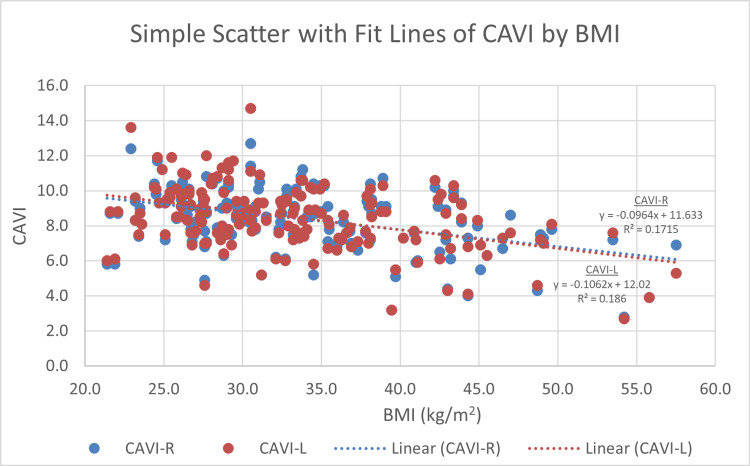
Scatter plot of right and left CAVI scores by body mass index. CAVI: cardio-ankle vascular index; CAVI-R: right cardio-ankle vascular index; CAVI-L: left cardio-ankle vascular index.

**Figure 6:**
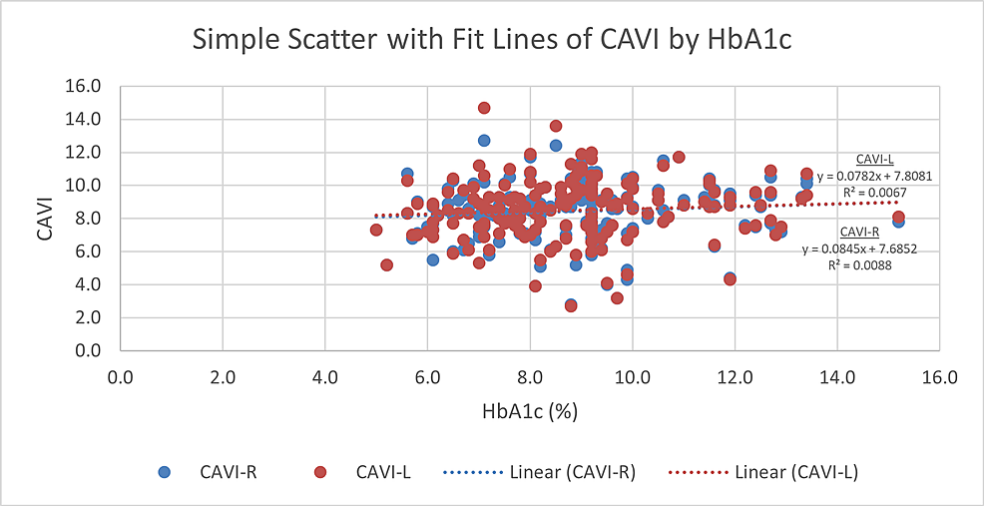
Scatter plot of right and left CAVI scores by HbA1c. CAVI: cardio-ankle vascular index; CAVI-R: right cardio-ankle vascular index; CAVI-L: left cardio-ankle vascular index; HbA1c: hemoglobin A1c.

**Figure 7:**
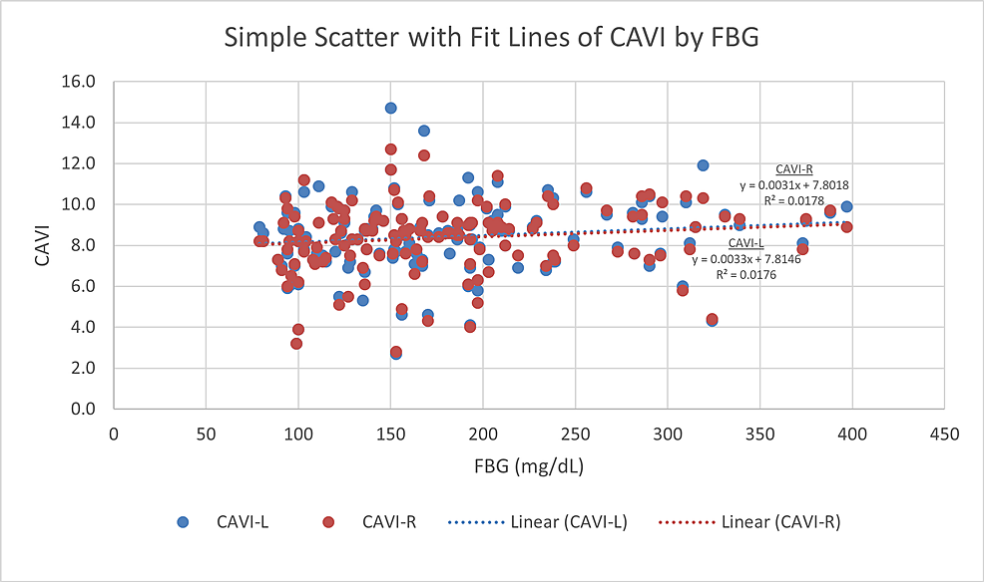
Scatter plot of right and left CAVI scores by fasting blood glucose. FBG: fasting blood glucose; CAVI: cardio-ankle vascular index; CAVI-R: right cardio-ankle vascular index; CAVI-L: left cardio-ankle vascular index.

Right CAVI and left CAVI measures display a significant correlation with age. The more senior participants have statistically significant higher CAVI scores (r = 0.417, p < 0.001 and r = 0.405, p < 0.001, respectively) (Figure 4). BMI displays an inverse correlation with both right and left CAVI readings (r = −0.411, p < 0.001 and r = −0.425, p < 0.001, respectively) (Figure 5). For blood glucose levels, although the findings are not significant, right and left CAVI scores are higher in participants with higher HbA1c and FBG (Figures 6, 7). Table 3 describes the correlation of right and left CAVI readings with the study variables.

**Table 3:** Clinical characteristics of the study sample and bivariate analysis with right CAVI and left CAVI (n = 188). BMI: body mass index; LDL: low-density lipoprotein; HDL: high-density lipoprotein; BUN: blood urea nitrogen; CAVI: cardio-ankle vascular index; HbA1c: hemoglobin A1c; FBG: fasting blood glucose. * P-value of 0.05 or less is statistically significant.

Variable	Right CAVI	Left CAVI
Physical constitution		
Age (years)	r = 0.417 (p < 0.001*)	r = 0.405 (p < 0.001*)
Weight (kg)	r = −0.345 (p < 0.001*)	r = −0.341 (p < 0.001*)
Height (cm)	r = 0.118 (p = 0.124)	r = 0.150 (p = 0.048*)
BMI (kg/m^2^)	r = −0.411 (p < 0.001*)	r = −0.425 (p < 0.001*)
Blood chemistry		
HbA1c (%)	r = 0.092 (p = 0.228)	r = 0.082 (p = 0.284)
FBG (mg/dL)	r = 0.146 (p = 0.084)	r = 0.138 (p = 0.104)
Total cholesterol (mg/dL)	r = −0.147 (p = 0.084)	r = −0.113 (p = 0.186)
LDL (mg/dL)	r = 0.079 (p = 0.358)	r = −0.061 (p = 0.476)
HDL (mg/dL)	r = −0.114 (p = 0.182)	r = −0.108 (p = 0.208)
Triglycerides (mg/dL)	r = −0.112 (p = 0.19)	r = 0.052 (p = 0.548)
Hemodynamics		
Left systolic blood pressure (mmHg)	r = −0.14 (p = 0.855)	r = −0.29 (p = 0.766)
Left diastolic blood pressure (mmHg)	r = 0.025 (p = 0.742)	r = 0.043 (p = 0.575)
Left mean arterial pressure (mmHg)	r = −0.07 (p = 0.86)	r = 0.26 (p = 0.16)
Left pulse pressure (mmHg)	r = −0.031 (p = 0.683)	r = −0.022 (p = 0.778)

## Discussion

In this study, we aimed to assess the relationship of different metabolic measures with hemodynamic measures. Owing to the fact that CAVI is not the standard modality of arterial wall stiffness assessment in KFHU, it was difficult to find an adequate number of pre-existing charts for CAVI readings for patients with T2DM. Thus, a larger sample size could not be achieved with the available charts. The study is also limited by the lack of longer-term follow-up with the sample population.

The significant findings of the study are the relationship between gender, age, and history of hypertension with CAVI measurements. CAVI readings are inversely correlated with BMI. As for glucose levels, we found an insignificant positive correlation of CAVI scores with HbA1c and FBG.

Arterial wall stiffness is defined as dysfunction of the arterial elastic capacity that leads to abnormal increases in the systolic blood pressure and decreases in diastolic blood pressure. A stiff arterial wall will require more pressure from the heart to overcome arterial dysfunction and stiffness. Increased heart pressure will increase the arterial pressure and therefore affect the volume by reducing it since the arterial wall cannot recoil properly. Clinical manifestations of arterial stiffness include stroke and left ventricle enlargement as a result of increased blood pressure. Chronic exposure to elevated levels of blood pressure could result in heart failure, a condition in which the heart cannot meet the body’s demands [7].

Multiple studies concluded that there is a correlation between increased arterial stiffness in patients with T2DM [8,9]. Also, the presence of diabetes mellitus increased the risk of microvascular and macrovascular complications such as arterial stiffness [10-14]. Pulse wave velocity (PWV) is a known tool for estimating arterial stiffness. A study done by Elias et al. based on a prospective analysis of the Maine-Syracuse study to link T2DM and arterial stiffness came to an interesting effect of diabetes on the PWV and thus, the arterial stiffness, in which they found that PWV readings were significantly higher in individuals with T2DM than healthy individuals. In addition to this conclusion, they also compared diabetics with controlled and uncontrolled glycemic levels. The uncontrolled group had more than three times the risk of having elevated PWV than the controlled group. Furthermore, comparing the uncontrolled to the non-diabetic group found more than eight times the risk of having a high PWV, which lead ultimately to linking diabetes to arterial stiffness, and more so in uncontrolled diabetes. In addition, patients with T2DM were found to have elevated pulse pressure (PP) and it has been found that elevated PP is predictive of future cardiovascular attacks [15-17].

The main measurement tool used in the data collected was CAVI, which is a noninvasive test used to estimate arterial stiffness by measuring brachial and ankle blood pressure, PP, and mean arterial pressure for both right and left sides. It is well understood that certain metabolic diseases can affect the health of the arterial wall, leading to arterial dysfunction and arterial stiffness [15]. CAVI measurement has been reported as a predictor of prognosis of the patients with cardiovascular disease [18]. Also, it has been reported that CAVI measurements are highly associated with blood glucose and HbA1c levels. Patients with elevated FBG and HbA1c tend to have higher CAVI measurements in relation to the normal population. Moreover, previous studies demonstrated that high levels of HbA1c are associated with increased lipid profile such as triglyceride and low-density lipoprotein levels, which eventually could precipitate the cardio-vascular events and nephropathies [19,20].

The results of this study display further emphasis on the relationship between glucose levels and arterial wall health.

## Conclusions

Although not statistically significant, CAVI had a linear association with blood glucose readings. This study yields that there is evidence suggesting that abnormally elevated blood chemistry, blood glucose levels, and blood pressure measurements are linked with higher CAVI scores, demonstrating their important effect on arterial wall stiffness while showing that CAVI can be used in predicting the prognosis of arterial wall health in patients with diabetes.

More studies are needed with a larger sample size to identify the major risks of developing arterial stiffness, and longer follow-up with the patients' data recording could yield more accurate results.
